# P2X7 Integrates PI3K/AKT and AMPK-PRAS40-mTOR Signaling Pathways to Mediate Tumor Cell Death

**DOI:** 10.1371/journal.pone.0060184

**Published:** 2013-04-02

**Authors:** Shu Bian, Xiaofeng Sun, Aiping Bai, Chunqing Zhang, Linglin Li, Keiichi Enjyoji, Wolfgang G. Junger, Simon C. Robson, Yan Wu

**Affiliations:** 1 Department of Gastroenterology, Provincial Hospital Affiliated to Shandong University, Jinan, People’s Republic of China; 2 Department of Medicine, Transplant Institute, Harvard Medical School, Boston, Massachusetts, United States of America; 3 Department of Surgery, Beth Israel Deaconess Medical Center, Harvard Medical School, Boston, Massachusetts, United States of America; University Paris Sud, France

## Abstract

**Background:**

Extracellular adenosine triphosphate (ATP) functions as a novel danger signal that boosts antitumor immunity and can also directly kill tumor cells. We have previously reported that chronic exposure of tumor cells to ATP provokes P2X7-mediated tumor cell death, by as yet incompletely defined molecular mechanisms.

**Methodology/Principal Findings:**

Here, we show that acute exposure of tumor cells to ATP results in rapid cytotoxic effects impacting several aspects of cell growth/survival, leading to inhibition of tumor growth *in vitro* and *in vivo*. Using agonist and antagonist studies together with generation of P2X7 deficient tumor cell lines by lentiviral shRNA delivery system, we confirm P2X7 to be the central control node transmitting extracellular ATP signals. We identify that downstream intracellular signaling regulatory networks implicate two signaling pathways: the known P2X7-PI3K/AKT axis and remarkably a novel P2X7-AMPK-PRAS40-mTOR axis. When exposed to high levels of extracellular ATP, these two signaling axes perturb the balance between growth and autophagy, thereby promoting tumor cell death.

**Conclusions:**

Our study defines novel molecular mechanisms underpinning the antitumor actions of P2X7 and provides a further rationale for purine-based drugs in targeted cancer therapy.

## Introduction

Extracellular adenosine triphosphate (ATP) functions as a danger signal, which is operational through type 2 purinergic (P2) receptors. ATP initiates intracellular signaling cascades that participate in many pathophysiological processes e.g. proliferation, differentiation, apoptosis, inflammation, and metabolism [1]–[3]. There are two P2 receptor families that associate with ATP and other nucleoside triphosphates and diphosphates: seven P2X receptors (P2X_1−_7) that are ATP-gated ion channels and eight G protein-coupled P2Y receptors (P2Y_1, 2, 4, 6, 11–14_). Different P2 receptors have differential agonist affinity/specificity whereby modulating different cellular functions [4], [5].

High levels of pericellular ATP are thought to exert antitumor activity through various molecular mechanisms [2], [6]–[8]. First, it has been shown that ATP promotes antitumor immune responses by: 1) enhancing dendritic cell-primed tumor specific CD8 T cell cytotoxicity [9], [10]; 2) acting as a “find-me” signal to phagocytes [11]; 3) induction of IL-1β release by monocytes [12], [13]; 4) skewing of T helper (Th) cells towards type 1 (Th1) and type 17 (Th17) cells which are thought to promote antitumor immunity [14]–[16]; and 5) limiting immunosuppressive activity by induction of regulatory T cell (Treg) apoptosis [17], [18]. Many of these effects appear mediated via activation of P2X7 receptor but the direct signaling pathways implicated are uncertain.

Additionally, high ATP levels have also been reported to exhibit direct cytotoxicity on many types of tumor cells such as prostate cancer, melanoma, glioma, and colon cancer cells [1], [19]. Among the fifteen P2 receptors, five subtypes specifically P2X_5_, P2X7, P2Y_1_, P2Y_2_, and P2Y_11_ (human exclusive), have been associated with the direct tumor-killing functions of ATP, but the precise molecular mechanisms remain somewhat unclear [1].

More recently, our laboratory has shown that high levels of extracellular ATP (eATP) effectively inhibit proliferation and induce apoptosis/necrosis of tumor cells suggesting anti tumor potential of purine-based drugs [15], [20]. In these studies, tumor cell death occurred as a result of the combined actions of ATP and its purine derivatives e.g. adenosine diphosphate (ADP) and adenosine. However, it is quite challenging to maintain eATP at high levels in the tumor microenvironment for long periods because of the abundant expression of ATP-degrading enzymes chiefly CD39/ENTPD1 (nucleoside triphosphate diphosphohydrolase-1) by tumor-associated vascular, immune and stromal cells, and/or tumor cells per se [2], [21].

Although we have linked eATP-elicited tumor cell death to P2X7 purinergic receptor [20], the intracellular signaling cascades of eATP-P2X7 are not fully described. Signal transduction networks are very complex and blockade of one component often disrupts key negative feedback mechanisms resulting in aberrant activation of compensatory pathways. As such, further defining the components of cytotoxic ATP-initiated purinergic signaling pathways is critical for the development of effective cancer therapies. For example, the mammalian target of rapamycin (mTOR) has been proven to be a promising cancer target and several rapamycin-related compounds (rapalogs) are now in various stages of clinical development as anticancer agents [22], [23]. However, rapalog exposure unfortunately disrupts crucial negative feedback modulations causing subsequent activation of phosphatidylinositol-3-OH kinase (PI3K)/protein kinase B (AKT) and/or PI3K-mitogen-activated protein kinase (MAPK) pathways and ultimately leading to chemoresistance [23], [24]. Therefore, a better understanding of the complexity of purinergic signaling (inclusive of negative feed-back loops) will be required to understand more clearly the biological consequences of inhibiting specific components of these cellular regulatory networks, as in cancer therapy.

We show here that brief exposure of tumor cells to ATP is able to efficiently induce cell death (including reduction of cell growth and induction of autophagy), mediated largely via P2X7. We further delineate the downstream components of ATP-P2X7 signaling, namely the known links to the PI3K/AKT axis [25] and a novel axis of AMP kinase (AMPK)-proline-rich AKT substrate of 40 KDa (PRAS40)-mTOR. These two seemingly independent signaling axes appear to act synergistically in response to tumoricidal ATP-P2X7 signals to thereby elicit maximal tumor cell death by disrupting the balance between cell growth and autophagy, without disturbing the negative feedback regulations as those seen with rapalogs. Our data provide further evidence as well as molecular basis for the utility of purine-based drugs as adjunctive agents in cancer therapy.

## Materials and Methods

### Ethics Statement

All mice were kept in a pathogen-free, temperature-controlled room with alternating 12hr dark/light cycles at the Beth Israel Deaconess Medical Center (BIDMC) and were negative for tests of Virology, Bacteriology, Infectious Disease PCR, Parasitology, and Pathology. Animal Experimentation Protocols were reviewed and approved by the Institutional Animal Care and Use Committees (IACUC) of BIDMC. The approved protocol number is #139-2009.

### Mice

Eight to twelve week old male C57BL/6 wild type mice were purchased from Taconic (MA).

### General Reagents/Antibodies

LY294002, rapamycin and compound C were purchased from EMD Corp Biosciences (Brookfield, WI); okadaic acid, carbenoxolone, Z-VAD-fmk, necrostatin-1, BAPTA-AM and thapsigargin were from Tocris Bioscience (Ellisville, MO); all other chemicals were from Sigma-Aldrich (St. Louis, MO). Cell culture media were purchased from ATCC and all other culture reagents from Invitrogen (Carlsbad, CA). Lentiviral shRNA (Short hairpin RNA) empty vector and constructs were purchased from the MISSION^®^ RNAi library of Sigma-Aldrich and the viral power kit and packaging cell line 293FT were obtained from Invitrogen.

Rabbit anti-P2X_7_ antibody (#APR-004) was obtained from Alomone labs (Jerusalem, Israel); β-actin (AC-15, #ab6276) from Abcam (Cambridge, MA); phospho-AKT (S473) (#9271), phospho-PRAS40 (T246) (#2997), Phospho-AMPKα (T172) (#2535), Phospho-AMPKβ (S108) (#4181), phospho-ACC (S79) (#3661), phospho-mTOR (S2481) (#2974), phospho-S6K (T389) (#9205), phospho-S6 (S235/236) (#2211), phospho-4E-BP1 (T37/46) (#2885), AKT (#9272), PRAS40 (#2691), AMPKα (# 2603), AMPKβ (#4150), ACC (#3676), mTOR (#2972), S6K (#9202), S6 (#2317), 4E-BP1 (#9644), and LC3B (#2775) from Cell Signaling Technology (Danvers, MA); HRP-conjugated goat anti-mouse and donkey anti-rabbit IgG and the SuperSignal West Femto Maximum Sensitivity Substrate reagents (#PI-34096) were from Thermo Scientific (Rockford, IL).

### Tumor Cell Lines

Syngeneic C57BL/6 murine MCA38 colon cancer cells (a gift of Dr. Nicholas P. Restifo, National Cancer Institute) were provided by Dr. Alan B. Frey at New York University School of Medicine [26]–[28], and mouse melanoma cell line B16/F10 was from American Type Culture Collection (ATCC, Manassas, VA). Cells were also tested for *Mycoplasma* and other infections by mouse IMPACT III PCR Profile via RADIL (Columbia, MO) and were maintained, as previously described [15], [20].

### Assessment of Cell Viability and Proliferation

Cells (7.5×10^3^) were seeded into 96-well plates and cultured for 24 hr. Cells were then pulse-treated with ATP, BzATP, UTP, or thapsigargin for different times, replaced with fresh culture media, and grown for additional 16–24 hr. Cell viability was evaluated using Cell Counting Kit-8 (CCK-8, Dojindo Molecular Tech. Inc., Rockville, MD) that measures the activity of cellular dehydrogenases (correlating with cell proliferation), as previously established [20], [29].

### In Situ Cellular Analysis

Cells (7.5×10^3^) were seeded into Corning 3603 Black 96-well plates and grown for 24 hr before exposed to ATP for a short period of time. 16–24 hr later, cell growth was evaluated using the Celigo Cytometer (Cyntellect, Inc., San Diego, CA). Brightfield images of live cells were captured using the Celigo Cell Counting application as described previously [20].

### Real-time and Dynamic Monitoring of Cell Growth (Proliferation and Viability)

These were performed using the xCELLigence RTCA MP System (Roche Diagnostics, Indianapolis, IN) that non-invasively quantifies adherent cell proliferation and viability using an electronic readout called impedance (Cell Index) in real-time, according to the manufacturer’s instructions. Cells (1.5×10^4^) were seeded in 96X E-plates (#06472451001; Roche Diagnostics) containing microelectronic sensor arrays and left in tissue culture hood for 30 min at room temperature before inserting the plates on the RTCA MP station in a humidified incubator under an atmosphere of 5% CO_2_-95% air at 37°C. Cell growth were immediately monitored up to 24 hr, at which point plates were taken out and cells were treated with ATP. Cells were then continuously monitored for additional 48 hr. Data were analyzed with RTCA software. The Cell Index values (indicating adhesion, spreading, proliferation and viability of the cells) were plotted against growing times.

### Clonogenic Assay

This was done as previously described with slight alterations [30]. Briefly, MCA38 colon cancer cells (1×10^6^) were grown in 60-mm culture dishes overnight to 60–70% confluency. Cells were pulse treated with ATP, trypsinized and resuspended in culture media. Then, 500 or 1000 cells were re-seeded into a 100-mm culture dish and cultured for 12 days. Fresh media were replaced every 3 days. On day 12, culture media were removed and colonies were stained with 4 ml of Clonogenic Reagent (0.25% 1,9-dimethyl-methylene blue in 50% Ethanol) at room temperature for 45 min. Then Clonogenic Reagent was removed by washing three times with PBS and blue colonies were counted and photographed using a digital Nikon camera.

### Antagonist-treatment Experiments

Cells were pre-incubated with antagonists compound C, okadaic acid, LY294002, rapamycin, KN-62, suramin, carbenoxolone, Z-VAD-fmk, necrostatin-1, or BAPTA-AM for 30 to 60 min before exposed to pulse treatment with ATP or control vehicle DMSO. The final concentration of DMSO added to the cells was <0.1%. No effect of DMSO was observed.

### Western Blotting

Cells (5×10^5^) were seeded into 6-well plates in culture media for 8 hr and then replaced with FBS-free starvation media. The next day, cells were treated with freshly prepared compounds in starvation media for various periods of time. Cells used for examination of cellular autophagy were grown and treated in regular culture media containing 10% FBS as serum-deprivation induces autophagy. Treatments were stopped immediately by washing the cells with ice-cold PBS three times. Cells were then lysed in ice-cold modified-RIPA buffer (50 mM Tris-HCl, pH 7.4; 1% NP-40; 0.25% sodium deoxycholate; 150 mM NaCl) supplemented with Complete Proteinase Inhibitor Cocktails (Roche Diagnostics) and Phosphatase Inhibitor Cocktails (Sigma-Aldrich). The lysates were sonicated briefly on ice and centrifuged at 14,000 rpm for 10 minutes at 4°C. The measurement of protein concentrations and detailed procedures of immunoblotting were described previously [29], [31].

### Reverse Transcription-PCR (RT-PCR)

Total RNA were extracted and purified from cells using an RNeasy kit (Qiagen, Germany). Reverse transcription was conducted on 1 µg of total RNA using ABI Prism TaqMan reverse transcription reagents (Cat#204054, Applied Biosystems, Foster City, CA). The specificity of primers for murine P2 receptors and β-actin has been validated; sequences and PCR conditions were used exactly as published previously [20], [29], [32]. Sequences of specific primer pairs for P2X7(a) and P2X7(k) variants and PCR conditions were as previously described [33]. All primers were obtained from Invitrogen.

### Generation of P2X7 Deficient Cell Lines

MCA38 or B16/F10 cells were infected separately with an empty shRNA vector control (pLKO.1-puro), or four different mouse P2X7 shRNA (#1: NM-011027.1-105s1c1, TRCN0000068572; #2: NM-011027.1-610s1c1, TRCN0000068570; #3: NM-011027.1-876s1c1, TRCN0000068568; and #4: NM-011027.1-1368s1c1, TRCN0000068571) lentiviral transduction particles (Invitrogen), according to the manufacturer’s instructions. Recombinant lentiviral particles were produced by transient transfection of 293FT cells according to standard protocol (Invitrogen). Briefly, 6×10^6^ of 293FT cells were seeded in a 100-mm culture dish in DMEM without antibiotics. The next day, cells reaching 90–95% confluence were cotransfected with 3 µg of shRNA plasmids and 9 µg of ViraPower™ Packaging Mix (an optimized proprietary mix of three plasmids, pLP1, pLP2, and pLP/VSVG from Invitrogen) using lipofectamine 2000 (Invitrogen). After 16 hr culture medium was switched to regular growth medium and cells were incubated for additional 48 hr. Conditioned cell culture media containing recombinant lentiviral particles were harvested, filtered through a 0.45 µm syringe filter, aliquoted, and used immediately or stored at -80°C. MCA38 or B16/F10 cells were infected with above cell culture supernatant containing lentiviral particles in the presence of polybrene (8 mg/ml) for 24 hr. These cells were replaced with regular growth media and cultured for additional 48 hr to allow the expression of selection marker, and then selected with puromycin (3 µg/ml for MCA38 cells and 1.5 µg/ml for B16/F10 cells) to generate stable cell lines encoding empty vector shRNA and P2X7 shRNA, for at least 10 days. The selected cell lines were tested to confirm diminished P2X7 expression, by Western blot analysis.

### Ethidium Bromide Uptake Assay

This assay was performed as previously published [34] with slight modifications. Briefly, cells (1.5×10^4^) were seeded into 96-well plates. 24 hr later, cells were washed once with HBSS containing Ca^2+/^Mg^2+^ and then incubated with 1.27 µM ethidium bromide (a cell impermeable organic dye) in the absence or presence of ATP (2.5 mM for MCA38 and 5 mM for B16/F10) for different times, followed by whole cell fluorescence measurement (in arbitrary units of fluorescence, AUF) at 544/610 nM excitation/emission using the SoftMax Pro software on a SpectraMax M5 Microplate Reader (Molecular Devices, Sunnyvale, CA). Background fluorescence was subtracted to obtain the ATP-elicited fluorescence.

### Intracellular Nucleotide Measurements

Intracellular ATP, ADP, and AMP were determined by high-performance liquid chromatography (HPLC) analysis as described [35]–[37] with modifications. 2.5×10^5^ cells were seeded into 12-well plates in culture media for 8 hr and then replaced with FBS-free starvation media. 24 hr later, cells were treated with ATP in starvation media for various times, followed by washing with ice-cold HBSS five times to remove excess extracellular ATP. Some wells were lysed with 200 µl of protein lysis buffer for protein concentration measurement; and some wells were harvested with 600 µl of HBSS containing 5% 8 M perchloric acid and then subjected to three frozen-thaw cycles. Lysates were scraped, transferred to a 1.5 ml Eppendorf tube, pulse sonicated on ice, and then stored at −80°C for subsequent HPLC analysis using a Waters 484 system (Waters Corporation, Milford, MA) as previously described [35]–[37].

### Tumor Inoculations

This was preformed as previously described with slight modifications [15], [38]. Briefly, cells (2×10^6^) were seeded into 100-mm dishes. The next day, immediately after ATP treatment (2.5 mM for 30 min), cells were washed with PBS, trypsinized, and resuspended in culture media. 2×10^5^ of tumor cells were injected s.c. into each flank of syngeneic 8–12 weeks old male C57BL/6 wild type mice. On day 14, the mice were euthanized and examined for tumor growth.

### Statistical Analysis

All data are represented as means ± SEM of values (obtained from three to six independent experiments in triplicates or quadruplicates). All statistical analyses were performed using the two-tailed Student’s *t*-test or the one-way analysis of variance (ANOVA) followed by the LSD (least significant difference) test when *F* was significant using SPSS 17 for Windows software. Significance was defined as *P<*0.05.

## Results

### Antitumor Actions of ATP: *in vitro* and *in vivo* Studies

In this study, we first sought to shorten ATP treatment time while still achieve efficient tumor-killing activity using two types of tumor cells: MCA38 colon cancer cells and B16/F10 melanoma cells, as in prior studies [20].

**Figure 1:**
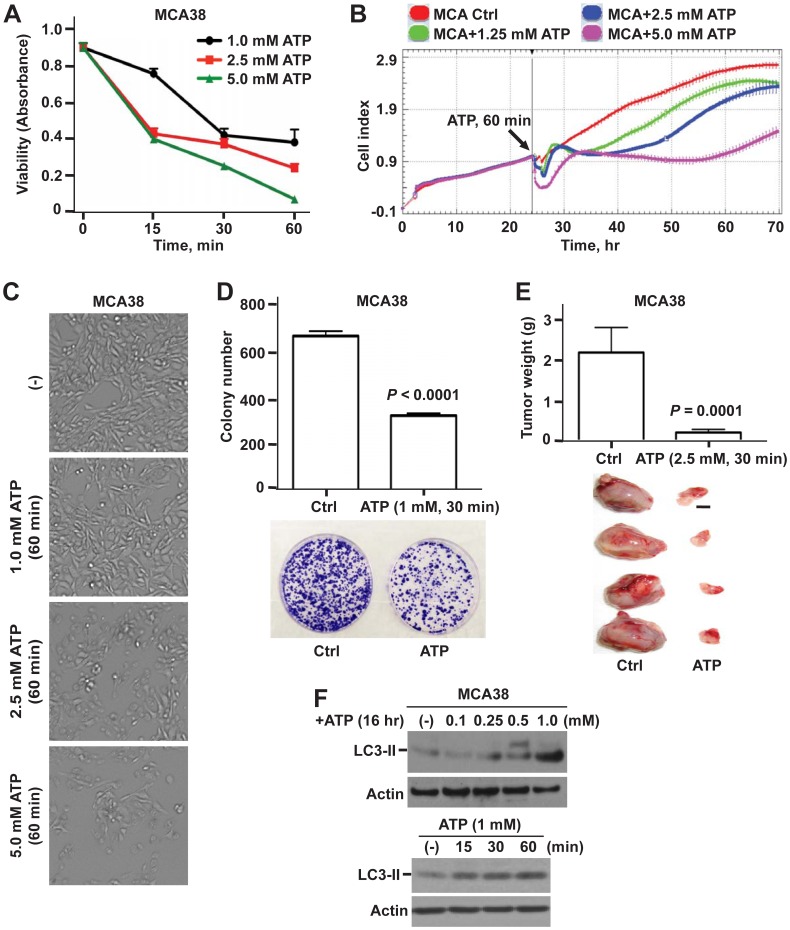
Antitumor effects of ATP on MCA38 colon cancer cells: *in vitro* and *in vivo* studies. A–C) Dose- and time-dependent responses of MCA38 cells to ATP cytotoxicity at 24 hr after treatment: cell viability/proliferation by Cell Counting Kit-8 (CCK-8) (A); real-time and dynamic monitoring of cell growth by xCELLigence instrument (B); and representative bright field images of live cells by Celligo Cell Counting application (C). **D)** Top, colony numbers of MCA38 cells on day 12 post ATP treatment. Bottom, representative images of clonogenic assay dishes. **E)** Top, tumor weight of C57BL/6 mice implanted with untreated (Ctrl) or ATP-treated MCA38 cancer cells on day 14 (n = 12 per group). Bottom, representative tumors on day 14. Scale bar, 0.5 cm. **F)** Western blots of LC3-II, a sensitive indicator for autophagy, using MCA38 cells treated with ATP at various doses and times as indicated. β-actin serves as a loading control. Error bars, mean ± SEM. Data represent three to six experiments.

As shown in Fig. 1A–E (MCA38 cells) and in Fig. S1A–C (B16/F10 cells), brief exposure of ATP (as short as 15 min) was able to effectively kill both types of tumor cells (albeit with differential potency), in a time- and dose-dependent manner. These findings are in accord with our prior findings using repeated and/or protracted exposure to ATP [20]. Cytotoxic ATP impacts many aspects of tumor cell growth/survival: viability and proliferation (Fig. 1A–C; Fig. S1A–C) as well as clonogenicity (Fig. 1D) *in vitro*; and *in vivo* tumor growth in C57BL/6 wild type mice (Fig. 1E). Strikingly, tumor cells responded to ATP cytotoxicity immediately, as evaluated by real-time measurement of tumor cell growth (Fig. S1B). Noteworthily, tumor cell death observed here was exclusively attributed to the action of ATP as ADP or adenosine showed no effects on tumor growth even at very high concentrations (up to 10 mM) for 1 hour (data not shown).

Moreover, tumor cell growth is tightly controlled by autophagy, which is a fundamental process to maintain cellular homeostasis and bioenergetics [39]. Induction of autophagy by manipulations of autophagic pathways has been shown to have broad antitumor potential (e.g. inhibiting xenograft tumor growth, reducing cancer metastases, and inducing regression of existing cancers) [40], [41]. As such, we next examined the effects of eATP on tumor cell autophagy. Light chain 3-II (LC3-II), a sensitive marker for autophagy, was used here. Autophagy is generally suppressed in the presence of serum. Herein, we observed that ATP markedly enhanced LC3-II levels in tumor cells growing in complete growth medium containing 10% serum, also in a time- and dose-dependent fashion (Fig. 1F; Fig. S1D).

These results are suggestive of links between tumor cell growth and autophagy that are co-responsive to cytotoxic ATP.

### Signaling Regulatory Networks of PI3K/AKT, AMPK and mTOR Impacted by ATP

ATP has been shown to activate mTOR signaling with co-occurring elevation of LC3-II levels in microglial cells [42]. mTOR is a well-known key player in many critical cellular processes such as proliferation, survival, autophagy, and metabolism [22], [43]. Moreover, PI3K/AKT and AMPK pathways are the two well-established positive and negative upstream arms of mTOR, respectively [44], [45]. It has been demonstrated that ATP induces depletion of nuclear phosphorylated AKT [25].

**Figure 2:**
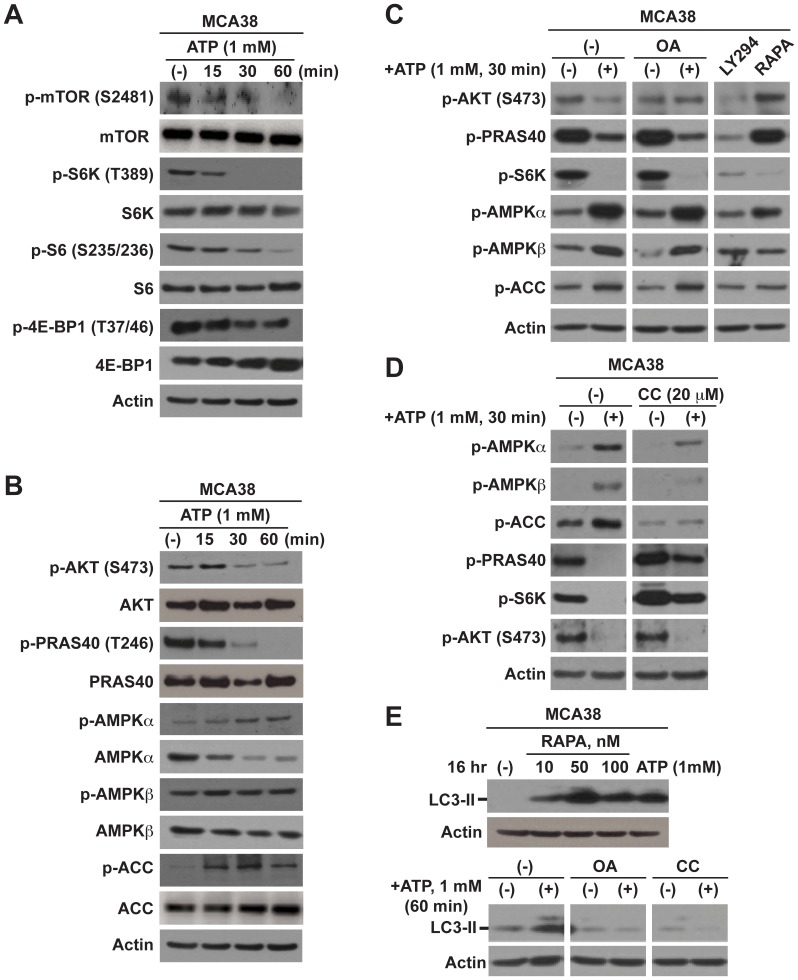
Intracellular mTOR, AKT, and AMPK signaling and induction of autophagy are impacted by cytotoxic ATP. A–B) Western blot analysis of signaling components of mTOR (A), AKT and AMPK (B), in MCA38 cells after ATP treatment at different times as indicated. Inhibition of mTOR and AKT with concurrent stimulation of AMPK signaling was noted. **C–D)** Delineation of ATP-elicited intracellular signaling cascades by blockade of certain component using specific inhibitors, determined by Western blotting. OA (okadaic acid): 100 nM; LY294 (LY294002): 50 µM; RAPA (rapamycin): 100 nM; CC (compound C): 20 µM. **E)** Impacts of pathway inhibitors on autophagy in MCA38 cells, as examined by Western blots of LC3-II. β-actin is the loading control. Data represent three to six experiments.

Hence, we first examined the phosphorylation of pathway components of mTOR (inclusive of mTOR, p70 S6 kinase (S6K), S6 ribosomal protein, and translation repressor protein 4E-BP1), PI3K/AKT (AKT and PRAS40), and AMPK (AMPKα, AMPKβ and acetyl-CoA carboxylase (ACC)) in response to eATP. Time- and dose-dependent decreases in phosphorylated levels of mTOR and PI3K/AKT signaling molecules concomitant with increases in AMPK cascade were noted (Fig. 2A and B; Fig. S2A). These effects occurred regardless of the cell growing conditions (serum-starvation medium or complete growth medium) (data not shown).

To further delineate regulatory networks for ATP-induced alterations in mTOR, PI3K/Akt and AMPK signaling, we performed the following experiments using pharmacologic inhibitors to these pathways. Phosphorylation of S6K, a sensitive and immediate downstream target of mTOR, was used to indicate changes in activation status of mTOR. First, as noted in Fig. 2C, okadaic acid (OA), a potent inhibitor of protein phosphatase 1 (PP1) and protein phosphatase 2A (PP2A) preventing p-Akt depletion, fully abolished ATP-mediated depletion of p-AKT whereas with no effects on depletion of its conventional target p-PRAS40 (Thr246). However, okadaic acid failed to reverse ATP-induced inhibition on mTOR and cell growth by (Fig. 2C). In parallel, inhibitor to PI3K (LY294002) could almost completely block basal phosphorylation of AKT-PRAS40-S6K (Fig. 2C). As expected, rapamycin (inhibitor of mTOR) suppressed S6K phosphorylation, to a similar extent as ATP (Fig. 2C). These data suggest that PI3K/AKT-PRAS40 is an upstream of mTOR in tumor cells under physiological conditions but not involved in ATP-induced mTOR inactivation.

Second, blockade of AMPK activation by compound C (CC) completely rescued ATP-elicited de-phosphorylation of S6K (Fig. 2D; Fig. S2B). Intriguingly, ATP-mediated depletion of p-PRAS40, (as suggested by its name proline-rich AKT substrate of 40 KDa) a well-recognized classical downstream target of AKT, was near wholly abolished by compound C, as similar to that of p-S6K (Fig. 2D; Fig. S2B). These data are implicative of a novel AMPK-PRAS40-mTOR axis responding to tumoricidal ATP.

Finally, we explored the links between mTOR signaling and tumor cell autophagy that are altered by eATP. Indeed, mTOR is thought to suppress autophagy [46]. We observed that rapamycin dramatically enhanced LC3-II levels in tumor cells, similar to ATP (Fig. 2E, top). Furthermore, okadaic acid or compound C could substantively abrogate LC3-II at basal levels or induced by ATP (Fig. 2E, bottom). However, okadaic acid or compound C alone failed to rescue ATP-elicited tumor cell death (Fig. S2C). Indeed, compound C treatment alone slightly but significantly reduced cell growth (*P* = 0.002; Fig. S2C). Moreover, blockade of either PI3K/AKT (by LY294002) or mTOR (by rapamycin), or both, was able to induce cell death but with much less potency when compared to ATP (Fig. S2C).

Taken together, these data indicate that cytotoxicity of ATP is mediated by two independent signaling pathways, namely AMPK-PRAS40-mTOR and PI3K/AKT, that synergistically perturb (otherwise tightly controlled) balance between growth and autophagy in tumor cells whereby eventually causing cell death. The inability to rescue ATP-evoked tumor cell death by blocking either pathway inhibition induced by ATP imply that upstream molecule(s) must exist for these two ATP-initiated signaling cascades that are urgently needed to be identified for targeted cancer therapy.

### P2X7 Receptor is the Control Nexus of ATP-elicited Tumor Cell Death

**Figure 3:**
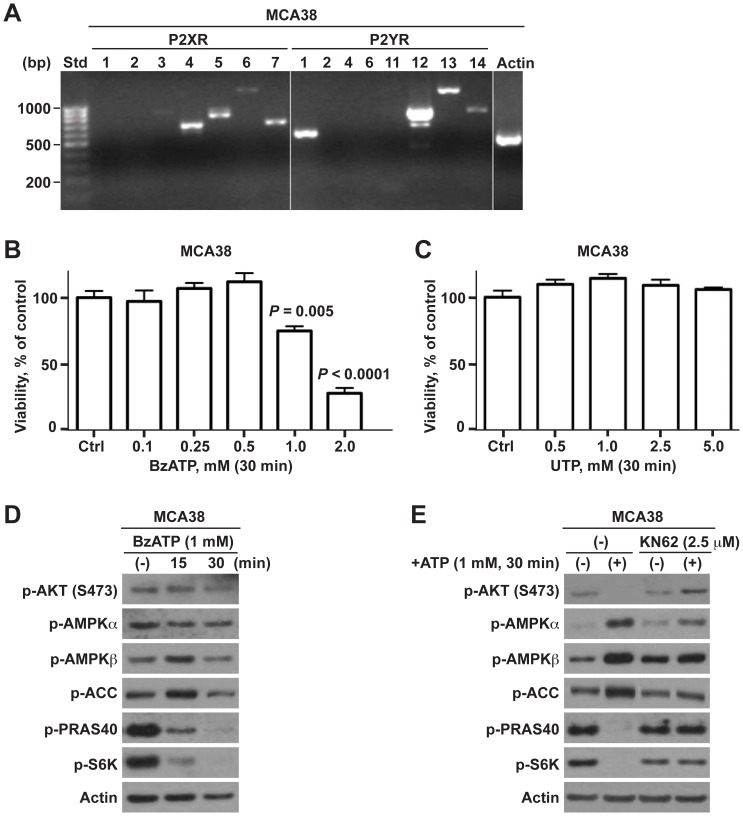
ATP-induced tumor cell death is modulated via P2X7-mTOR axis. **A)** Reverse transcription-PCR (RT-PCR) analysis of mRNA expression of P2 receptors in MCA38 cells. Size standards (Std) are shown in the left lane. **B–C)** Cell viability of MCA38 cells at 24 hr post treatment with BzATP (B) or UTP (C), as determined by CCK-8. Data are expressed as a percentage of untreated controls. **D)** Impacts of BzATP on AKT, AMPK, and mTOR signaling, as analyzed by Western blot. **E)** Blockade of P2X7 by antagonist KN62 abrogated ATP-induced inhibition on mTOR signaling in MCA38 cells, as evaluated by Western blotting. β-actin is shown as a loading control. Error bars, mean ± SEM. Data represent three to four experiments.

Therefore, we next focused on searching for the purinergic receptor(s) transmitting extracellular cytostatic ATP-mediated signals to these intracellular regulatory networks as alluded above. We first analyzed mRNA expression of P2 receptor panels in MCA38 cells using RT-PCR (data with B16/F10 cells have been published previously (ref)). MCA38 cells expressed mRNA transcripts for P2X_3_ (minimal), P2X4, P2X_5_, P2X_6_ (weaker), P2X7, P2Y_1_, and P2Y_12–14_ (Fig. 3A).

We have previously reported that antitumor function of ATP is, at least partially, mediated via P2X7. We then treated tumor cells with BzATP, a more potent prototypic P2X7 receptor agonist. As presented in Fig. 3, BzATP-triggered growth inhibition (Fig. 3B; Fig. S3A) and signaling alterations (Fig. 3D) of tumor cells near exactly recapitulates the same pattern observed with ATP. In contrast, UTP, an agonist of P2X_5_, P2Y_2_, P2Y_4_, and P2Y_6_, exhibited no effects on tumor cell growth (Fig. 3C). Moreover, the global P2 receptor antagonist suramin (inhibiting P2X_1–3_, P2X_5_, P2Y_1–2_, P2Y_6_, and P2Y_11–13_) was unable to relieve the suppressive actions of ATP on phosphorylation of AKT, PRAS40 and S6K (Fig. S3B) as well as tumor cell growth (data not shown), although it could block ATP-induced activation of AMPK signaling (Fig. S3B). In parallel, selective P2X7 receptor antagonist KN62 virtually counteracted ATP-mediated signaling alterations, in a time- and dose-dependent manner (Fig. 3E; Fig. S3C and D).

These findings are evident of P2X7 receptor as the upstream control node of ATP-initiated signaling regulatory networks.

### Knockdown of P2X7 Protects Tumor Cells from Antitumor Function of ATP

**Figure 4:**
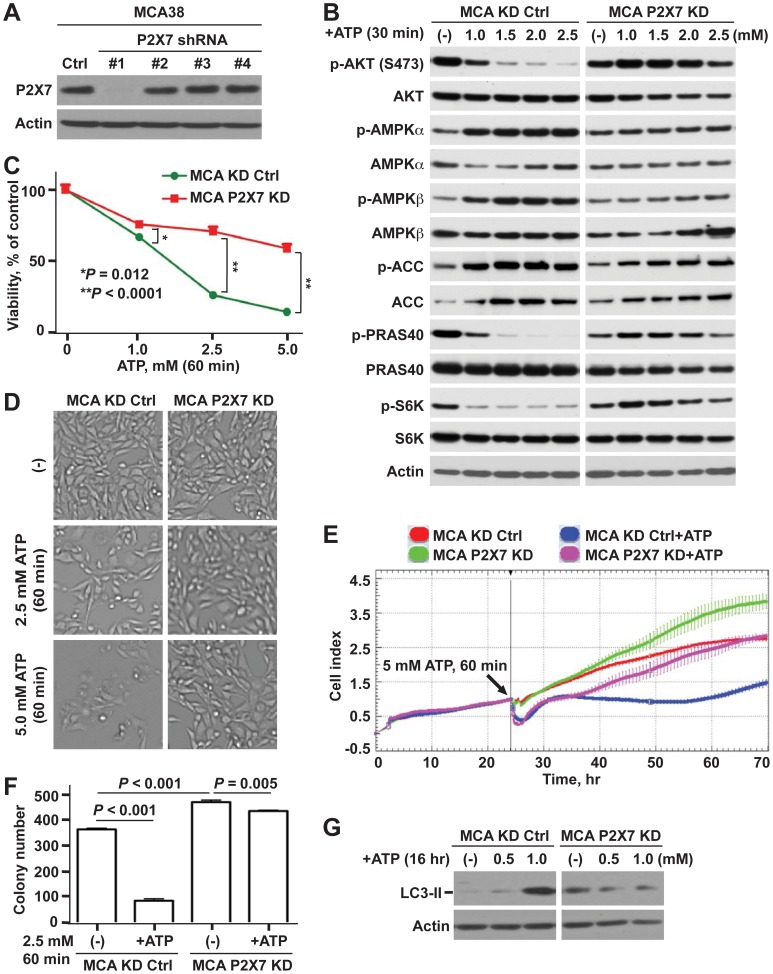
Knockdown of P2X7 decreases antitumor activity of ATP. **A)** Western blots for P2X7 using lysates from MCA38 cells after knocking down P2X7 using lentiviral shRNAs. **B–G)** Differential effects of ATP on control knockdown (KD Ctrl) and P2X7 deficient (P2X7 KD) MCA38 cells: AKT- and AMPK-mTOR signaling by Western blotting (B); cell viability by CCK-8 (C); representative live cell images by Celligo (D); real-time measurement of cell growth by xCELLigence (E); clonogenicity by colony formation assay (F); and autophagy by Western blotting (G). β-actin is a loading control. Error bars, mean ± SEM. Data represent three experiments.

However, KN62 is also a selective inhibitor of Ca^2+^/calmodulin-dependent protein kinases II (CaM kinases II), though exerting more potent inhibition on P2X7 receptor. CaM kinases II has been shown to involve in pathophysiological signaling to thereby modulate many fundamental cellular processes e.g. proliferation and apoptosis, and has been linked to ATP-provoked purinergic signaling in endothelial cells [47]. As such, we generated stable P2X7 deficient tumor cells using lentiviral shRNA method as detailed in Materials and Methods. Out of four P2X7 shRNAs, one effectively blocked P2X7 protein expression in both tumor cell lines (Fig. 4A; Fig. S4A). These are defined as P2X7 KD and used for all subsequent analyses.

Strikingly, P2X7 deficiency near fully deviated all cytostatic ATP-mediated effects on tumor cells: signaling regulatory networks (Fig. 4B; Fig. S4B), cell growth (Fig. 4C–E; Fig. S4C–E), colony-forming capacity (Fig. 4F), and autophagy (Fig. 4G; Fig. S4F). Further, knockdown of P2X7 conferred tumor cells with higher growth and clonogenic potential, as compared to control knockdown (Ctrl KD) cells (Fig. 4E and F; data not shown).

### Characterization of Extracellular ATP-elicited P2X7 Receptor Channel Functions

Next, we sought to assess the impact of tumoricidal concentrations of extracellular ATP on P2X7 receptor channel functionality.

**Figure 5:**
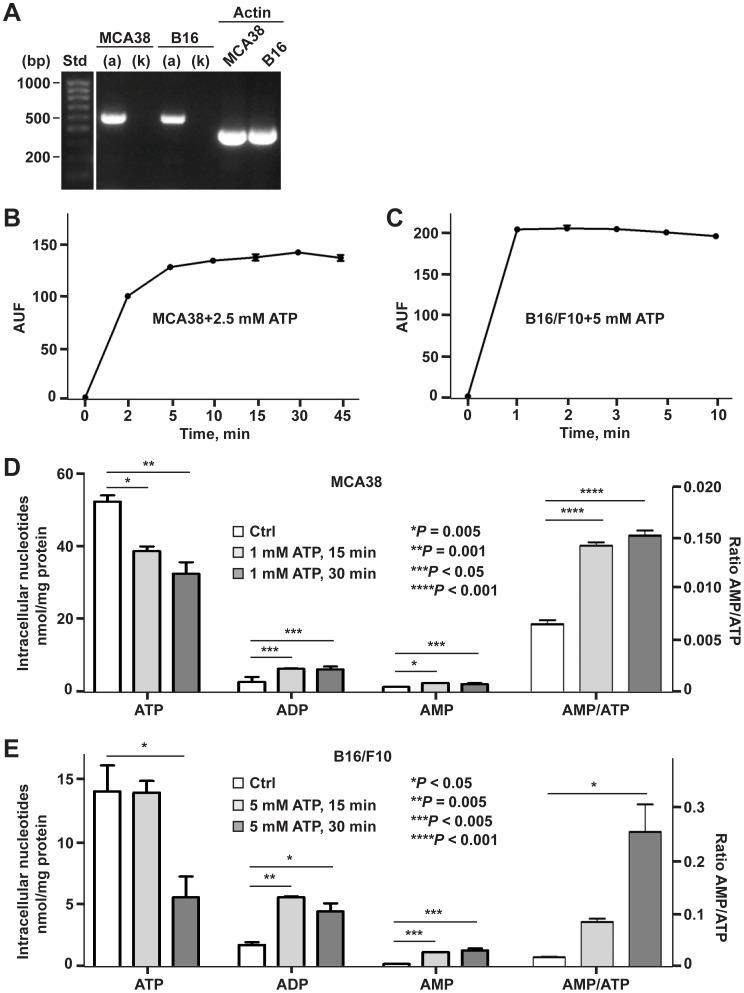
Characterization of P2X7 receptor function activated by tumoricidal ATP. **A)** Examination of P2X7(a) or P2X7(k) variant expression in MCA38 and B16/F10 cells by RT-PCR with specific primer pairs for P2X7(a) or P2X7(k). Size standards (Std) are shown in the left lane. **B–C)** ATP-stimulated ethidium bromide uptake in MCA38 (B) and B16/F10 cells (C). **D–E)** Effects of extracellular ATP on intracellular levels of ATP, ADP and AMP in MCA38 (D) and B16/F10 cells (E) were measured by HPLC. Values of MCA38 AMP concentrations are multiplied five fold for the sole convenience of plotting on the same axes. Error bars, mean ± SEM. Data represent three experiments.

First, we examined the expression of P2X7 splice variants P2X7(a) and P2X7(k) in tumor cells, as it has been recently reported that the P2X7(k) variant exhibits a much higher ligand sensitivity and transduces signals more efficiently than the P2X7(a) variant [33]. As shown in Fig. 5A, both MCA38 and B16/F10 cells only express the P2X7(a) variant at the mRNA level.

Second, P2X7 channel function, specifically non-selective pore activity, was evaluated using ethidium bromide uptake assays. We noted that ATP markedly stimulates ethidium bromide uptake in both cell lines (Fig. 5B and C).

Third, these demonstrated “porin” channels could facilitate efflux of intracellular adenine nucleotides thereby perturbing intracellular nucleotide levels. It is known that an increase in the AMP/ATP ratio is linked to the mechanism of AMPK activation. Therefore, we then measured levels of intracellular ATP, ADP and AMP by HPLC in tumor cells pulsed treated with ATP for 15 and 30 min. Substantial decreases in intracellular ATP levels were observed (Fig. 5D and E), concurrent with significant increases in intracellular ADP and AMP levels as well as AMP/ATP ratios (Fig. 5D and E). These observations are compatible with ATP-P2X7 triggered AMPK activation, as also noted above (Fig.2; Fig. S2).

Fourth, by performing antagonist studies, we then confirmed that PI3K/AKT and AMPK/mTOR signaling altered by P2X7 activation are not linked to other native pore-forming transporters (inclusive of pannexin- or connexin-type channels) nor reactive oxygen species (ROS), as previously reported [19], [48]. Neither carbenoxolone (a pharmacological inhibitor of pannexin- or connexin-type channels) nor N-acetyl-cysteine (a broad anti-oxidant) could substantively impact ATP-induced P2X7 signaling cascades (Fig. S5A).

We have previously demonstrated that ATP-P2X7 elicited tumor cell death is mediated via both apoptosis and necrosis [20], in keeping with fluxes in intracellular nucleotides, as also noted above. We now show that P2X7 activation can also induce tumor cell autophagy (Fig. 1F; Fig. 4G; Fig. S1D; Fig. S4F). Importantly, none of the specific pharmacological inhibitors (e.g. Z-VAD-fmk, a pan-caspase inhibitor or the necroptotic inhibitor necrostatin-1; various doses of compounds ranging from 12.5 to 200 µM were tested) when tested alone could counteract ATP-induced tumor cell death (Fig. S5B; data not shown).

### Calcium Signaling is not Involved in ATP-P2X7 Mediated Tumor-killing Activity

Given that P2X7 is a prototypic ligand-gated ion channel, the activation by high ATP levels often leads to a robust increase of Ca^2+^ influx into cells [42]. To determine whether the influx of Ca^2+^ is involved in ATP-evoked cellular effects, two experimental approaches were employed using two chemical compounds that differently alter cytosolic Ca^2+^ levels: BAPTA-AM, a selective cell-permeable calcium chelator to block intracellular Ca^2+^ stores, and thapsigargin (TG), a potent inhibitor of endoplasmic reticulum Ca^2+^-ATPase causing an immediate increase in cytoplasmic Ca^2+^ levels.

**Figure 6:**
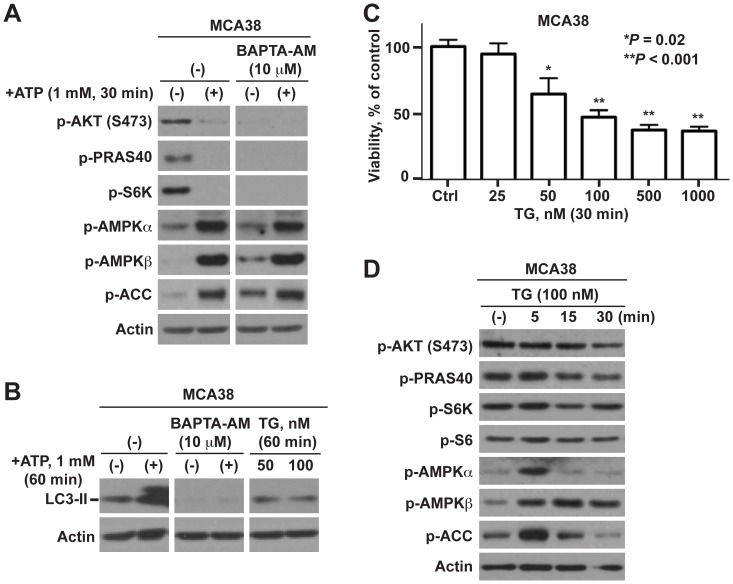
ATP-P2X7-mTOR elicited tumor cell death does not involve calcium signaling. **A)** Effects of BAPTA-AM on ATP/P2X7-initiated AKT, AMPK and mTOR signaling in MCA38 cells, as examined by Western blotting. **B)** Effects of BAPTA-AM and thapsigargin (TG) on autophagy as determined by Western blots of LC3-II. **C–D)** Impacts of thapsigargin (TG) on MCA38 cell growth by CCK-8 (C); and Akt, AMPK and mTOR signaling by Western blot analysis (D). β-actin is the loading control. Error bars, mean ± SEM. Data represent three experiments.

First, we found that BAPTA-AM failed to relieve ATP-induced mTOR inactivation and LC3-II increase (Fig. 6A and B; Fig. S6A). Instead, BAPTA-AM completely abolished basal levels of PI3K/AKT and mTOR signaling and LC3-II with no effects on AMPK signaling (Fig. 6A and B; Fig. S6A), associated with marked induction of cell death (Fig. S6B). This supports a role of Ca^2+^ in tumor cell growth under physiological conditions but not in cellular responses to cytotoxic ATP.

In contrast, such Ca^2+^-ATPase inhibitors as thapsigargin (TG), a potent antitumor agent can be used to induce autophagy in mammalian cells, were able to induce immediate cell death (Fig. 6C; Fig. S6C) and activation of AMPK pathway (Fig. 6D; Fig. S6D), but exerted no effects on PI3K/AKT and mTOR signaling transduction nor on autophagy (Fig. 6B and D; Fig. S6D). These data affirm that Ca^2+^ influx is not involved in the tumoricidal function of ATP having distinct molecular mechanism from the antitumor action of thapsigargin.

**Figure 7:**
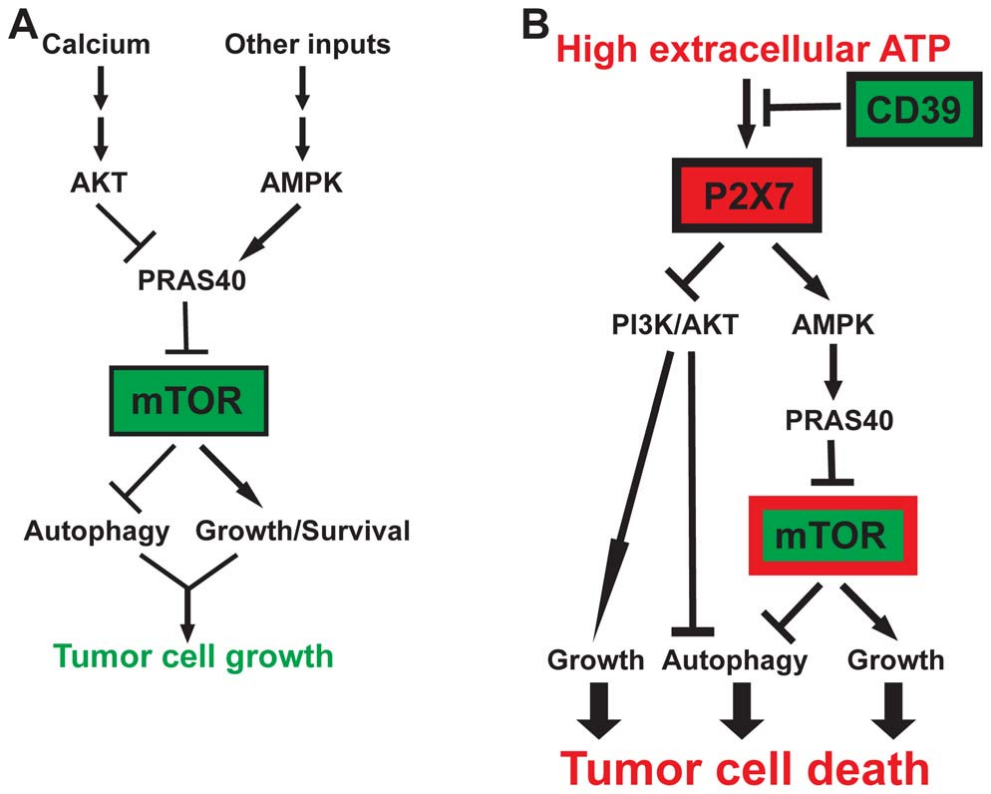
Schematic illustration of how PI3K/AKT, AMPK and mTOR regulatory networks distinctly control tumor cell growth in response to high levels of ATP. **A**) In the presence or absence of serum, tumor cell growth is dictated by cellular autophagy and growth/survival signals that are tightly controlled by PI3K/AKT and AMPK pathways convergent on mTOR by way of PRAS40. Physiological Ca^2+^ stores are absolutely required to maintain basal activation of the PI3K/AKT-PRAS40-mTOR signaling and cell growth. **B**) In a tumor microenvironment with pericellular ATP likely to be at high levels, activation of P2X7 on tumor cells leads to concurrent blockade of the mTOR signaling by way of AMPK-PRAS40 axis and the PI3K/AKT pathway. These are the two critical control nexuses for autophagy induction and growth inhibition that ultimately lead to tumor cell death. Ectonucleotidase CD39 protects tumor cells from antitumor activity of high levels of extracellular ATP [15], [20]. Pathway key: →: Stimulation; –ı: Inhibition.

In summary, we infer that high-levels of extracellular ATP act on one novel P2X7-AMPK-PRAS40-mTOR axis and one conventional P2X7-PI3K/AKT axis to induce autophagy and inhibit growth that ultimately result in tumor cell death (as illustrated in Fig. 7).

## Discussion

We have previously reported that protracted exposure to ATP, a putative danger signal enhances antitumor immunity, directly inhibits tumor cell growth and induces cell death, at least in part via activation of the purinergic receptor P2X7 [20]. In the present study, we further show that acute and self limited treatment with exogenous ATP is able to trigger a prompt cellular response that effectively hinders tumor growth, both *in vitro* and *in vivo*. More importantly, we provide a novel molecular mechanism that further links extracellular ATP-P2X7 signals to the intracellular mTOR pathway, one of the critical control nexuses of signaling transduction networks and used as a cancer therapeutic target [22], [23].

mTOR is a well-known sensor for extracellular nutrients and growth factors converging many crucial signals e.g. Ras/mitogen-activated protein kinase (MAPK) and PI3K/AKT to control tumor cell growth [43] and is known to regulate fundamental cellular functions inclusive of proliferation/growth, survival, migration, adhesion, autophagy, and metabolism [22]. The regulation networks of mTOR are very complex.

It has recently been shown that PRAS40 is a raptor-interacting protein inhibiting S6K1 phosphorylation and cell growth and AKT-mediated phosphorylation of PRAS40 prevents its suppression on mTOR [44]. Activation of P2X7 by ATP has been shown to cause a rapid depletion of nuclear phosphorylated AKT in non-small cell lung cancer cells A549 [25]. In addition, AMPK, a key player in maintaining energy homeostasis that is activated in response to cellular stress, is a negative upstream regulator of mTOR signaling [45]. Extracellular ATP could also activate AMPK, albeit independent of P2X7 in endothelial cells [47]. Intriguingly, distinct from the conventional mTOR modulation networks, we herein identify a novel AMPK-mTOR regulatory axis by way of de-phosphorylating Thr246 of PRAS40, the classical AKT substrate, in parallel with the previously-shown PI3K/AKT pathway, in response to tumoricidal ATP-P2X7 signaling (as illustrated in Fig. 6). Instead, PRAS40 is not the downstream component of ATP-P2X7-PI3K/AKT cascade. Moreover, we further note that in tumor cells under physiological conditions, PI3K/AKT and AMPK converge on PRAS40 to inversely modulate mTOR activation whereby tightly controlling the balance of cell growth and autophagy to govern tumor cell growth. *This is the first article to report the de-phosphorylation of PRAS40 at Thr246 by AMPK, which is operational as an upstream suppressor of mTOR*.

Furthermore, we show that tumoricidal ATP concentrations trigger non-selective pore-forming activity, characteristic of P2X7 activation. However, the noted ATP-P2X7-induced blockade of mTOR and PI3K/AKT signaling associated with tumor cell death is not regulated via pannexin- or connexin type channels nor by varied levels of mitochondrial ROS production, as previously reported in prior studies [19], [48]. The observed process in our studies also appears independent of the more well established P2X7 activation-elicited influx of Ca^2+^, such as seen in microglial cells and macrophages [42]. These latter observations are in line with prior studies showing that ATP triggered P2X7-mediated human leukemia cell death is independent of increased Ca^2+^ influx [49]. However, basal levels of Ca^2+^ stores do appear essential for the maintenance of PI3K/AKT-PRAS40-mTOR activation and tumor cell growth under normal growing conditions.

We further are able to link activation of AMPK, a well-known classical energy sensor, with increases of intracellular AMP/ATP ratios; both are altered by high extracellular ATP levels. Additionally, mTOR has also been established as an intracellular ATP/energy sensor [50]. Interestingly, our observations of time- and dose-dependent alterations of mTOR signaling by extracellular ATP suggest the possibility that mTOR also might function as a sensor for extracellular ATP levels in the tumor microenvironment.

Moreover, experiments with both temporary blockade of P2X7 by non-competitive antagonists e.g. KN62 and stable knockdown by lentiviral P2X7 shRNA imply that tumoricidal activity of ATP is, to a very great extent, mediated through P2X7 receptor signaling. Importantly, P2X7-deficient tumor cells exhibit greater potential of growth and clonogenicity. This observation is in keeping with a previous article reporting anthracycline treated breast cancer patients with loss of function allelic mutations of P2X7 develop metastatic disease more rapidly [9]. Collectively, these results support the concept that the P2X7 receptor might also link high levels of extracellular ATP to intracellular signaling transduction networks thereby promoting tumor cell death.

It has been demonstrated that any P2X7 knockdown results in a compensatory increase in expression of P2X4, another purinergic receptor with similar function to P2X7 yet less investigated/understood [51]. We also observed an increased P2X4 expression in both types of P2X7 deficient tumor cells (data not shown) that might be responsive to cytotoxic ATP. We have excluded the possibility of involvement of other type 2 purinergic receptors viz. P2X_1–3_, P2X_5_, P2Y_1–2_, P2Y_4_, P2Y_6_, and P2Y_11–13_, in ATP-elicited tumor cell death noted here by performing studies using P2 receptor agonist/antagonist panels (Fig. 3; Fig. S3).

We have recently defined that both apoptosis and necrosis are linked to ATP-P2X7 promoted tumor cell death [20]. However, suppression of either apoptosis or necroptosis alone fails to attenuate ATP-elicited tumor cell death here. Indeed, we can further link P2X7 activation to induction of tumor autophagy.

These data suggest that ATP-induced tumor cell death involves combinational and concurrent cellular events including opening of porin channel, efflux of cytosolic adenine nucleotides, transduction of signaling cascades, induction of apoptosis/necrosis/autophagy, as well as inhibition of proliferation/survival. Hence, blockade of any single process alone is unable to counteract tumor-killing function of ATP.

This supposition could be supported by the experimental observations that only blockade of P2X7 (by pharmacologic inhibition or genetic knockdown) can substantively attenuate tumoricidal activity of ATP. In contrast, none of the down stream pathway inhibitors have comparable activities, despite near full abrogation of the respective signaling cascade (such as compound C, okadaic acid).

Ideally, to achieve maximal efficacy of cancer therapy, integration of all potential antitumor actions would be required. Implicated mechanisms (as for purinergic mediators) include ATP-mediated antitumor immune responses, direct induction of tumor cell death, and blockade of tumor angiogenesis. A good proof-of-concept example is the recent study conducted by Wang et al. demonstrating that co-targeting of tumor cells and elicitation of antitumor immune responses dramatically inhibited trastuzumab-resistant breast cancers [52].

The observed variable impacts of P2X7 activation on cell viability and growth remain controversial. Such divergent effects appear to be impacted by kinetics and levels of extracellular ATP fluxes as well as being highly cell-type dependent. Based on published literature, it seems that P2X7 activation at low level facilitates growth/survival of certain types of tumor cells [53] whereas the extracellular nucleotide at high level promotes cell death [1]. In this study, we focused on the molecular mechanism(s) underpinning the direct cytotoxic effects of high levels of ATP on tumor cells. Our data are also in agreement with the recent findings showing that extracellular ATP causes human colon cancer cell death [19].

One of the major issues in cancer therapy is that disruption of one signaling component often induces compensatory pathways because of negative feedback regulations (e.g. rapalogs [23], [24]) or loss of suppressors (e.g. PTEN [54]). These issues will ultimately lead to acquired resistance. Thus, combined targeting of multiple pathways, especially the upstream components, has been actively pursued and proven to be a better approach [55]. Hence, we suggest a more promising cancer target, P2X7, as the upstream component of a tumoricidal ATP signal transduction network in that activation of it leads to concurrent and efficacious blockade of both PI3K/AKT and mTOR pathways, without affecting escape mechanisms as those often seen with rapalogs nor PTEN expression (data not shown).

High levels of pericellular ATP have been observed in tumor tissues of tumor-bearing mice after radiation and chemotherapy [10], [56]. First, ATP released by such dying tumor cells, has been recently identified as a novel danger signal to enhance dendritic cell-primed tumor specific CD8 T cell cytotoxicity via activation of P2X7-NLRP3 inflammasome [9], [10]. Thus, ATP augments antitumor immune responses. Secondly, high levels of ATP efficiently kill tumor cells [20] that are further affirmed herein. Third, ATP also exerts direct cytotoxicity on vascular endothelial cells such as liver sinusoidal endothelial cells that could limit tumor angiogenesis, also in a dose- and time-dependent fashion [20].

Therefore, it is feasible to propose that ATP release from dying tumor cells evoked by immuno/chemo/radiotherapy could augment antitumor activity via a positive feedback reaction, and that co-altering the tumor microenvironment in such a fashion that would keep ATP levels high for a short period (15 to 60 minutes) would augment the efficacy of anti-cancer therapy.

CD39/ENTPD1, the dominant ectonucleotidase expressed by the vasculature and immune cells (including leukocytes, monocytes/macrophages, dendritic cells, B cells, Treg, natural killer (NK)/T, and type 17 effector T (Th17) cells), tightly controls extracellular ATP levels by hydrolyzing ATP to ADP and then AMP [2], [16], [21]. We have shown that vascular CD39 promotes tumor growth by scavenging extracellular ATP [20]. In addition, many types of tumor cells intrinsically express CD39 and ecto-5′-nucleotidase (or CD73; degrading AMP to adenosine) being able to generate the immunosuppressant adenosine per se, and thereby dampen effector cell function [2], [57]. The tumor cells used in this study (MCA38 colon cancers and B16/F10 melanoma cells) do not express CD39/CD73 [15], [20]. Moreover, our accumulating data also show that CD39 expression by vascular cells per se is an absolute requisite for tumor angiogenesis [15], [38], [58], and that Treg-associated CD39 and CD73 produces adenosine to impede antitumor immune responses [15], [59].

Recently, we have further demonstrated that blockade of CD39 enzymatic activity using a specific pharmacological inhibitor, POM-1 (Polyoxometalate-1), greatly abrogates tumor growth in transplanted tumor models [15]. This has been attributed, by us, to be mediated via: defective tumor angiogenesis, augmented antitumor function of NK cells, as well as enhanced tumor-killing activity of high levels of ATP [15], [20]. In parallel, modifications of purinergic signaling/CD39 as a promising target in cancer therapy have also been endorsed independently by other laboratories and discussed in detail in two recent articles [60], [61].

In summary, we describe novel purinergic suppression mechanisms involving AMPK-PRAS40 novel elements of the mTOR signaling network, which are operational with PI3K/AKT established pathways. Our studies affirm the therapeutic potential of purine-related drugs, which might be used to simultaneously target tumor cells and the host immune system, as well as tumor angiogenesis.

## Supporting Information

Figure S1 **Extracellular ATP is cytotoxic for B16/F10 melanoma cells. A–C)** Dose- and time-dependent responses of B16/F10 cells to ATP killing: cell viability/proliferation CCK-8 (A); real-time cell growth by xCELLigence (B); and representative live cell images by Celligo (C). **D)** Dose-dependent induction of autophagy by ATP in B16/F10 cells, as determined by Western blots of autophagy marker LC3-II. β-actin serves as a loading control. Error bars, mean ± SEM. Data represent three experiments.
(TIF)

Figure S2 **Time- and dose-dependent responses of AKT, AMPK and mTOR to ATP-mediated signaling responses in tumor cells. A)** Western blots for AKT, AMPK and mTOR pathway components post ATP treatment at various times and doses in B16/F10 cells (A). **B)** AMPK inhibitor compound C (CC) fully rescued ATP-induced mTOR inhibition in MCA38 cells in a dose-dependent manner, as examined by Western blotting. **C)** Effects of pathways inhibitors on MCA38 cell growth, as examined by CCK-8 and expressed as percentage of untreated controls. Data represent three to four experiments.
(TIF)

Figure S3 **P2 receptor agonist and antagonist studies.** **A)** B16/F10 cell viability at 24 hr post BzATP treatment, as determined by CCK-8. Data are normalized to untreated controls. **B)** Effects of suramin (100 µM,) on AKT, AMPK and mTOR pathways in MCA38 cells, as examined by Western blot analysis. **C–D)** P2X_7_ antagonist KN62 counteracted ATP-evoked signaling transduction of AKT, AMPK, and mTOR in MCA38 cells (C) and B16/F10 cells (D), in a dose-dependent manner, as evaluated by Western blotting. β-actin is the loading control. Error bars, mean ± SEM. Data represent three to four experiments.
(TIF)

Figure S4 **P2X_7_ deficient B16/F10 cells.** **A)** Knockdown of P2X_7_ in B16/F10 cells was validated by Western blotting. **B–F)** Differential effects of ATP on control and P2X_7_ KD B16/F10 cells: AKT- and AMPK-mTOR signaling by Western blotting (B); cell viability by CCK-8 (C); representative live cell images by Celligo (D); and real-time monitoring of cell growth by xCELLigence (E); and autophagy by Western blots of LC3-II (F). β-actin is used as the loading control. Error bars, mean ± SEM. Data represent three experiments.
(TIF)

Figure S5 **Assessment of carbenoxolone, N-acetyl-cysteine, Z-VAD-fmk, and necrostatin-1 on ATP-P2X7 induced signaling or tumor cell death.** **A)** Effects of carbenoxolone (CBX) and N-acetyl-cysteine (NAC) on ATP-initiated AKT, AMPK and mTOR signaling in MCA38 and B16/F10 cells, as examined by Western blot analysis. **B)** Effects of Z-VAD-fmk and necrostatin-1 on ATP-induced MCA38 cell death, as examined by CCK-8 and expressed as percentage of untreated controls. β-actin served as a loading control. Error bars, mean ± SEM. Data represent three experiments.
(TIF)

Figure S6 **Impact of calcium signaling on AKT, AMPK and mTOR signaling transduction and tumor cell growth.** **A)** Effects of BAPTA-AM on AKT, AMPK and mTOR signaling in B16/F10 cells, as analyzed by Western blotting. **B)** Effects of BAPTA-AM on MCA38 cell growth, as examined by CCK-8 and expressed as percentage of untreated controls. **C–D)** Impacts of thapsigargin (TG) on B16/F10 cell viability by CCK-8 (C); and AKT, AMPK and mTOR signaling by Western blot analysis (D). β-actin is shown as a loading control. Error bars, mean ± SEM. Data represent three experiments.
(TIF)
