# Supplementary figures and images for: P2X7 Integrates PI3K/AKT and AMPK-PRAS40-mTOR Signaling Pathways to Mediate Tumor Cell Death
Source: PLoS One. 2013 Apr 2;8(4):e60184. doi: 10.1371/journal.pone.0060184 (PMC3615040; doi:10.1371/journal.pone.0060184)

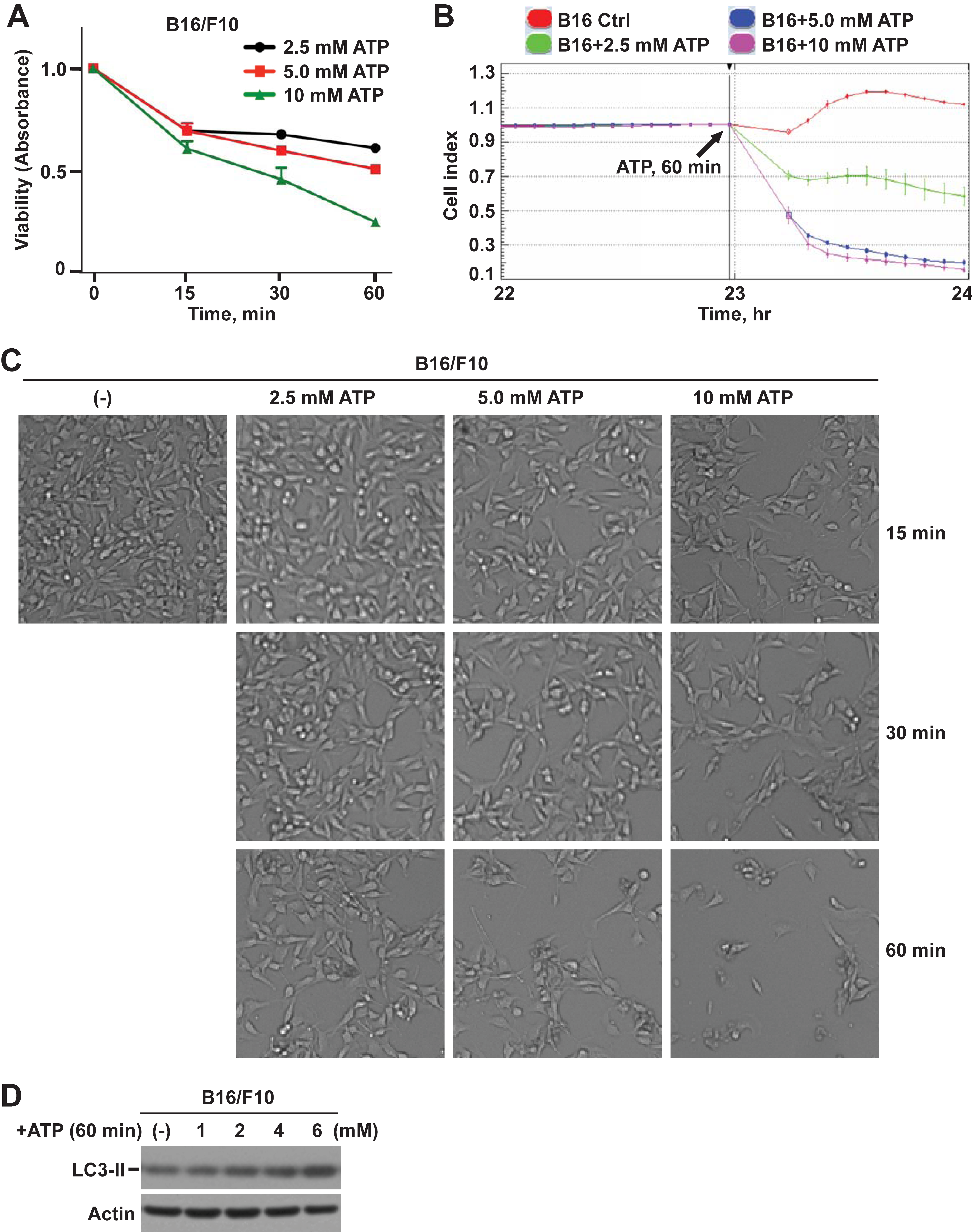

Supplement: Figure S1 — Extracellular ATP is cytotoxic for B16/F10 melanoma cells. A–C) Dose- and time-dependent responses of B16/F10 cells to ATP killing: cell viability/proliferation CCK-8 (A); real-time cell growth by xCELLigence (B); and representative live cell images by Celligo (C). D) Dose-dependent induction of autophagy by ATP in B16/F10 cells, as determined by Western blots of autophagy marker LC3-II. β-actin serves as a loading control. Error bars, mean ± SEM. Data represent three experiments. (TIF) [file pone.0060184.s001.tif]

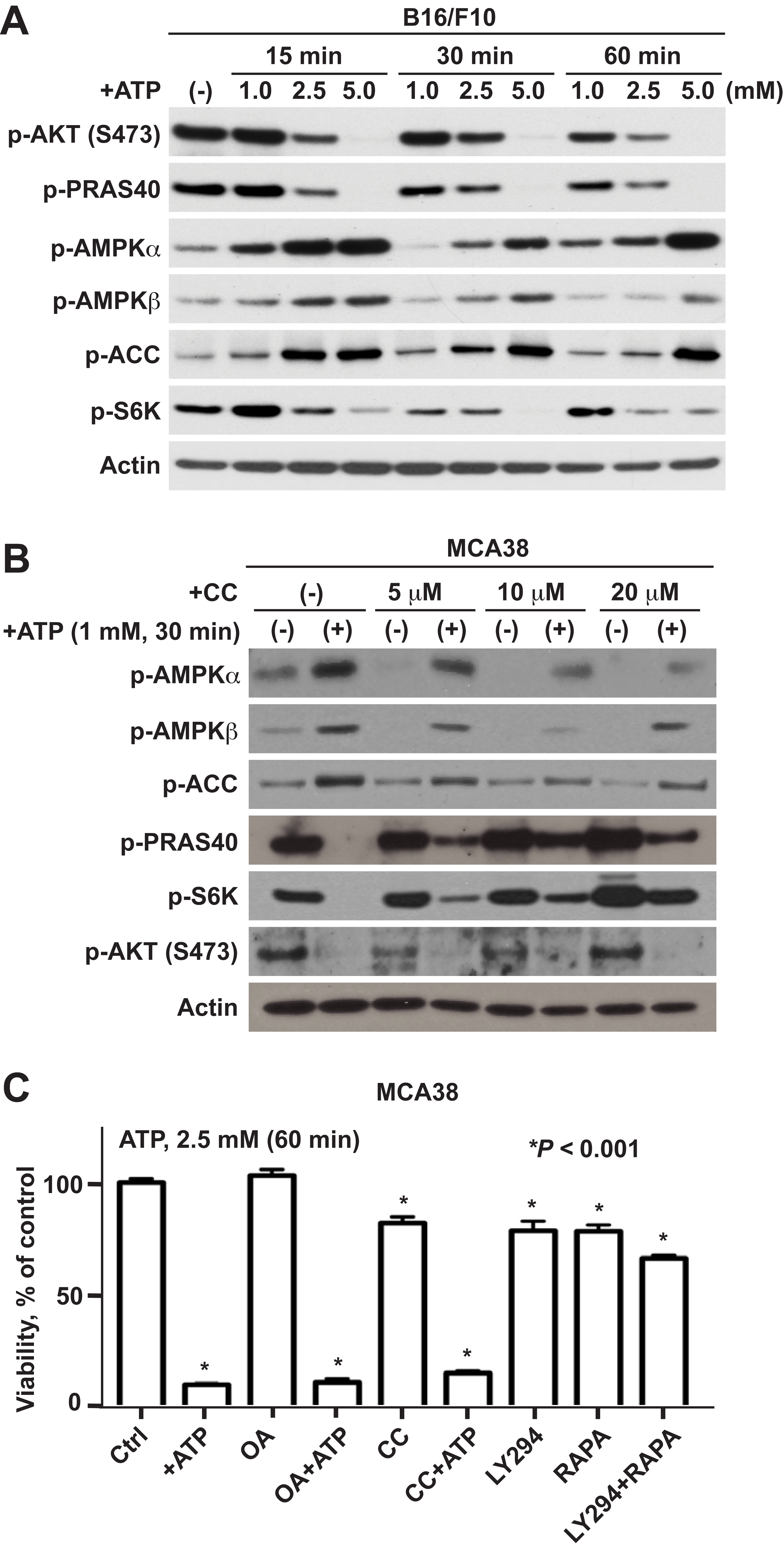

Supplement: Figure S2 — Time- and dose-dependent responses of AKT, AMPK and mTOR to ATP-mediated signaling responses in tumor cells. A) Western blots for AKT, AMPK and mTOR pathway components post ATP treatment at various times and doses in B16/F10 cells (A). B) AMPK inhibitor compound C (CC) fully rescued ATP-induced mTOR inhibition in MCA38 cells in a dose-dependent manner, as examined by Western blotting. C) Effects of pathways inhibitors on MCA38 cell growth, as examined by CCK-8 and expressed as percentage of untreated controls. Data represent three to four experiments. (TIF) [file pone.0060184.s002.tif]

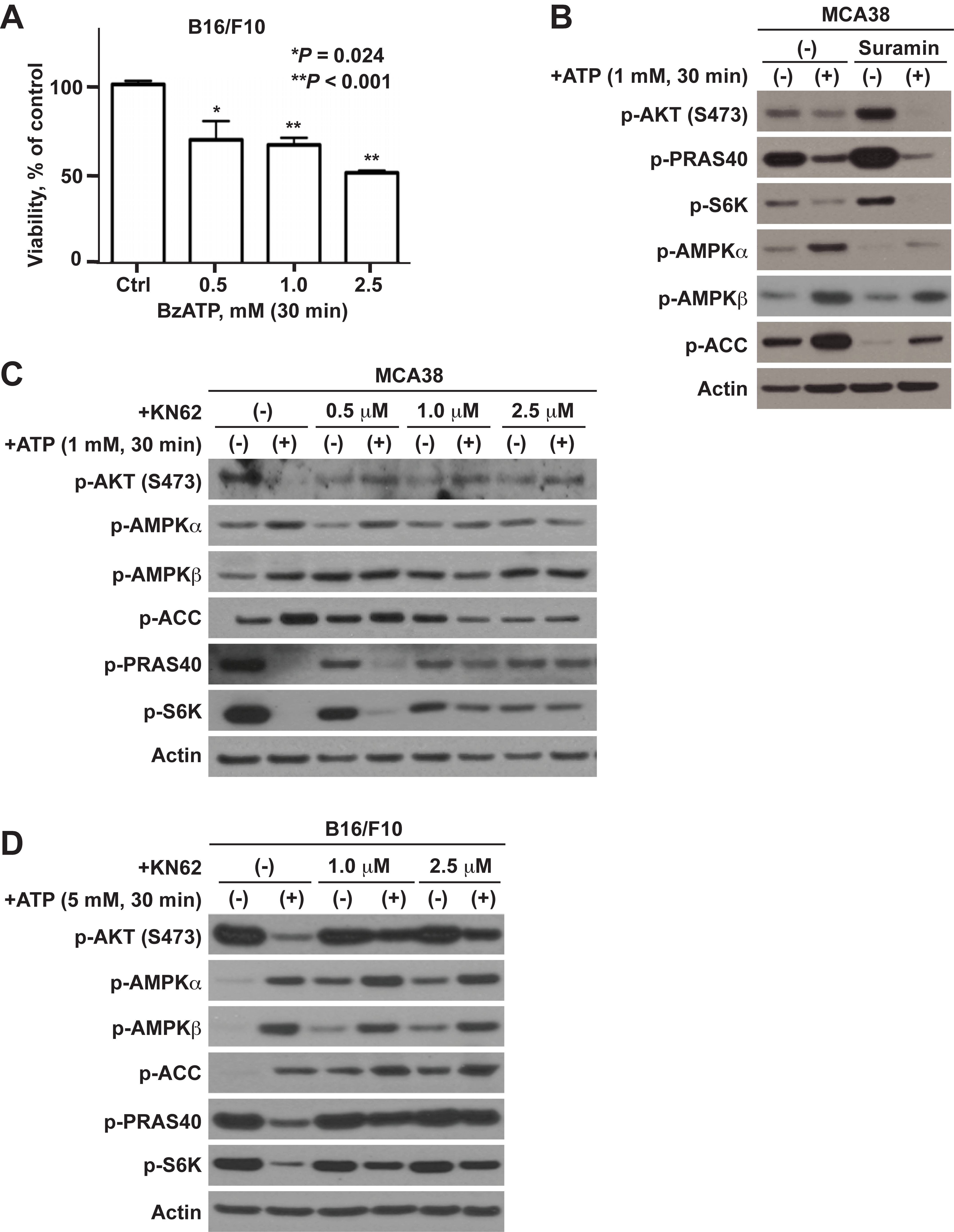

Supplement: Figure S3 — P2 receptor agonist and antagonist studies. A) B16/F10 cell viability at 24 hr post BzATP treatment, as determined by CCK-8. Data are normalized to untreated controls. B) Effects of suramin (100 µM,) on AKT, AMPK and mTOR pathways in MCA38 cells, as examined by Western blot analysis. C–D) P2X7 antagonist KN62 counteracted ATP-evoked signaling transduction of AKT, AMPK, and mTOR in MCA38 cells (C) and B16/F10 cells (D), in a dose-dependent manner, as evaluated by Western blotting. β-actin is the loading control. Error bars, mean ± SEM. Data represent three to four experiments. (TIF) [file pone.0060184.s003.tif]

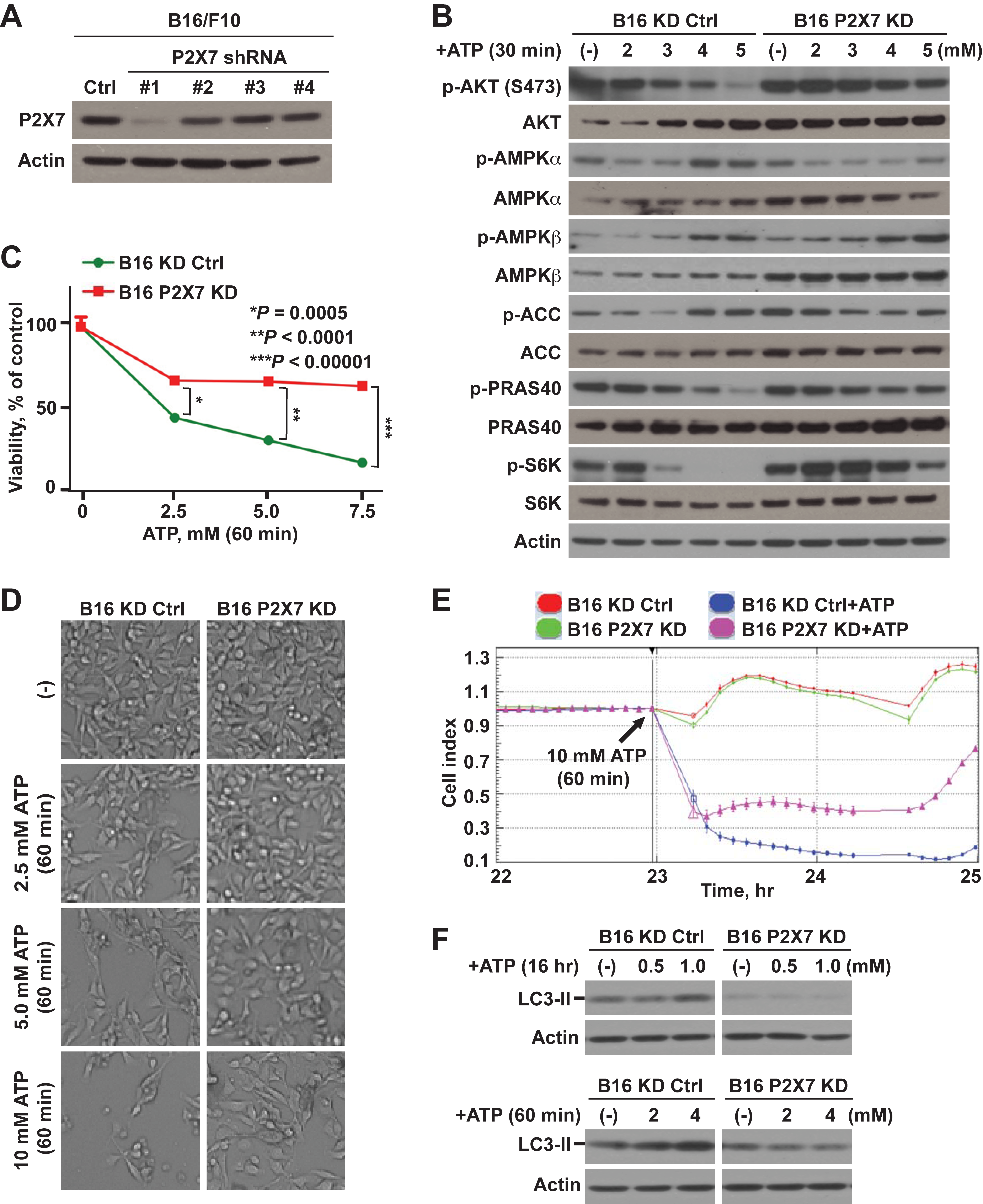

Supplement: Figure S4 — P2X7 deficient B16/F10 cells. A) Knockdown of P2X7 in B16/F10 cells was validated by Western blotting. B–F) Differential effects of ATP on control and P2X7 KD B16/F10 cells: AKT- and AMPK-mTOR signaling by Western blotting (B); cell viability by CCK-8 (C); representative live cell images by Celligo (D); and real-time monitoring of cell growth by xCELLigence (E); and autophagy by Western blots of LC3-II (F). β-actin is used as the loading control. Error bars, mean ± SEM. Data represent three experiments. (TIF) [file pone.0060184.s004.tif]

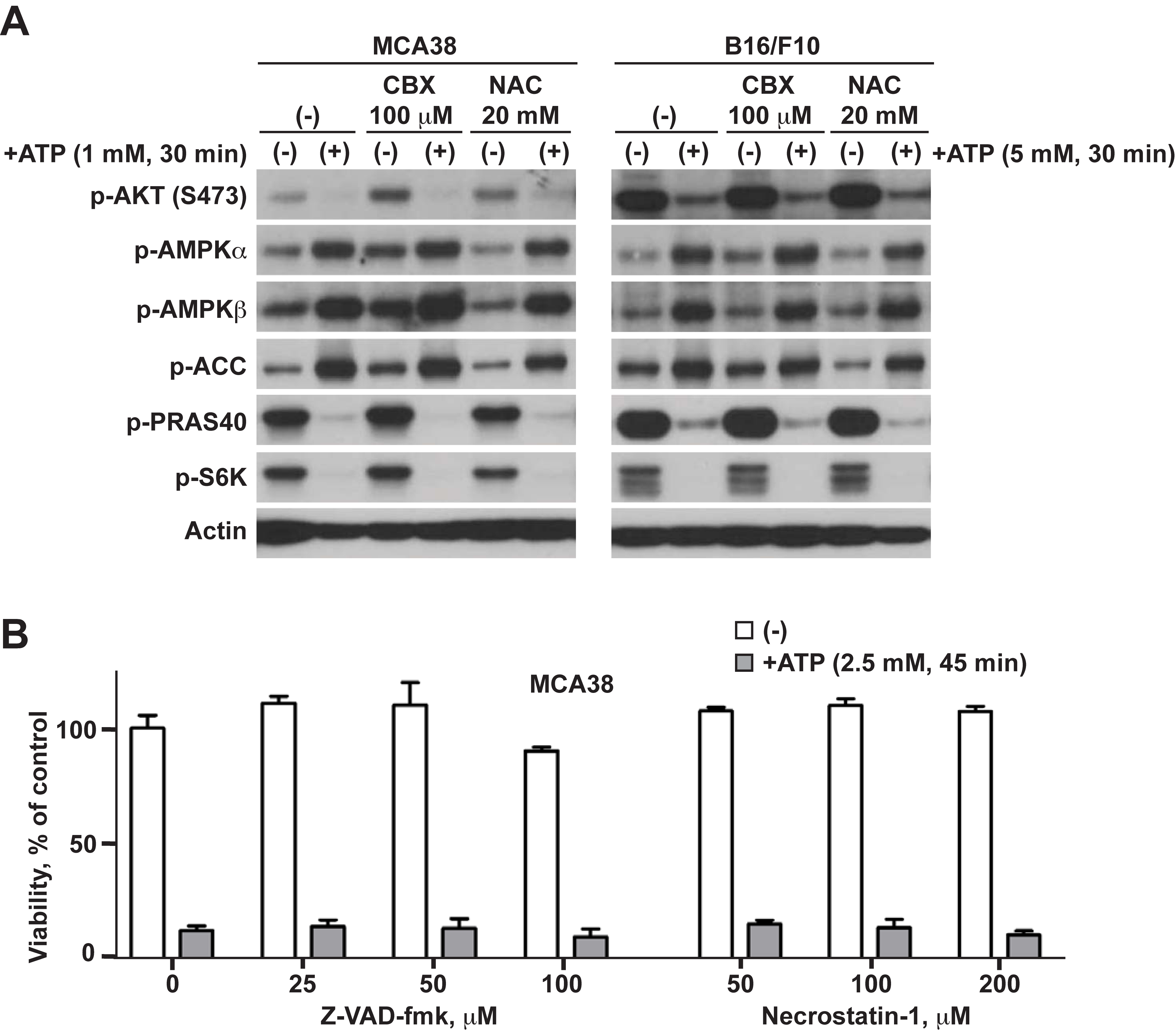

Supplement: Figure S5 — Assessment of carbenoxolone, N-acetyl-cysteine, Z-VAD-fmk, and necrostatin-1 on ATP-P2X7 induced signaling or tumor cell death. A) Effects of carbenoxolone (CBX) and N-acetyl-cysteine (NAC) on ATP-initiated AKT, AMPK and mTOR signaling in MCA38 and B16/F10 cells, as examined by Western blot analysis. B) Effects of Z-VAD-fmk and necrostatin-1 on ATP-induced MCA38 cell death, as examined by CCK-8 and expressed as percentage of untreated controls. β-actin served as a loading control. Error bars, mean ± SEM. Data represent three experiments. (TIF) [file pone.0060184.s005.tif]

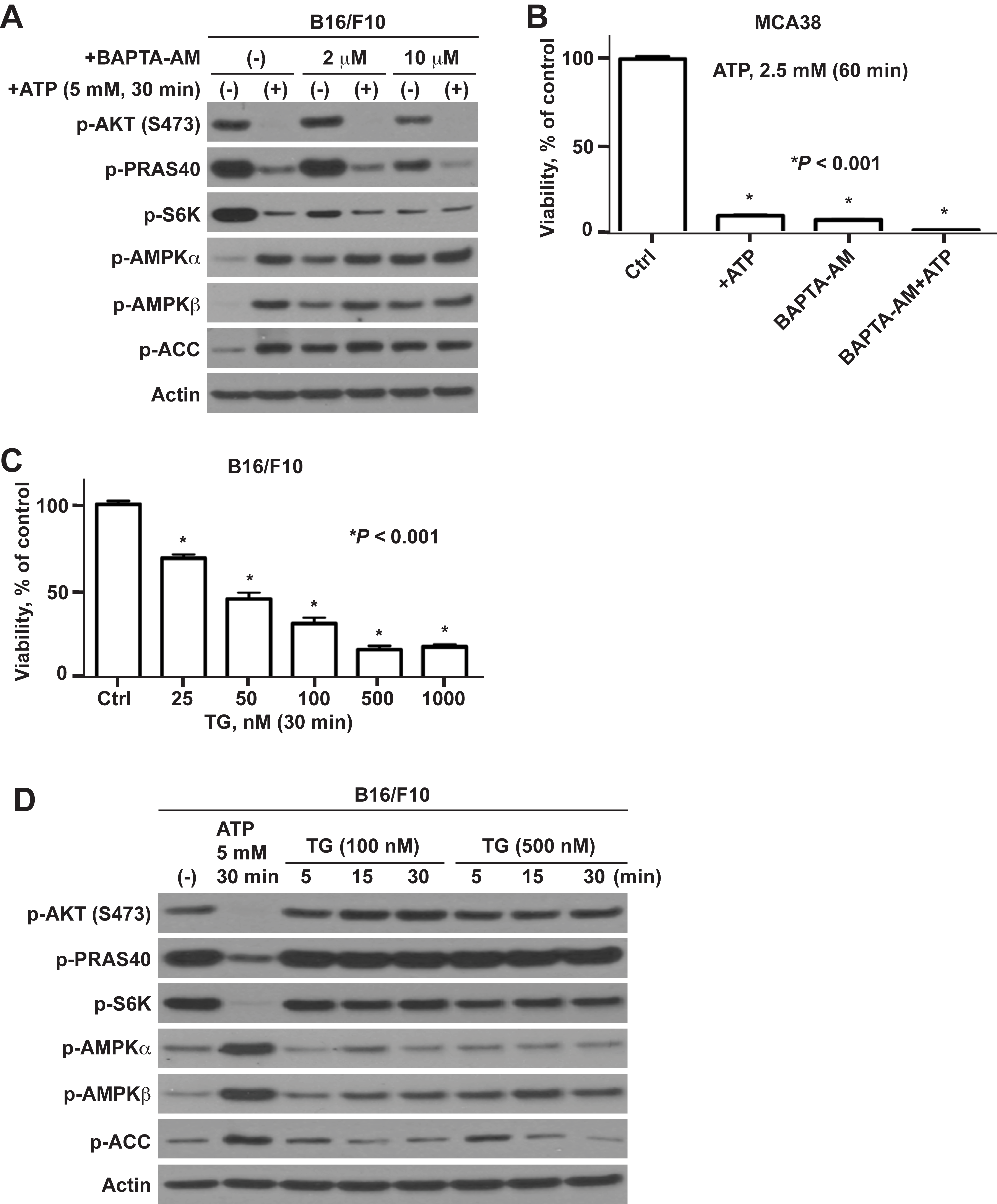

Supplement: Figure S6 — Impact of calcium signaling on AKT, AMPK and mTOR signaling transduction and tumor cell growth. A) Effects of BAPTA-AM on AKT, AMPK and mTOR signaling in B16/F10 cells, as analyzed by Western blotting. B) Effects of BAPTA-AM on MCA38 cell growth, as examined by CCK-8 and expressed as percentage of untreated controls. C–D) Impacts of thapsigargin (TG) on B16/F10 cell viability by CCK-8 (C); and AKT, AMPK and mTOR signaling by Western blot analysis (D). β-actin is shown as a loading control. Error bars, mean ± SEM. Data represent three experiments. (TIF) [file pone.0060184.s006.tif]
